# Changes in Influenza Activities Impacted by NPI Based on 4-Year Surveillance in China: Epidemic Patterns and Trends

**DOI:** 10.1007/s44197-023-00134-z

**Published:** 2023-08-03

**Authors:** Jing Tan, Lijun Liang, Ping Huang, Abrar A. Ibrahim, Zhongzhou Huang, Wei Zhao, Lirong Zou

**Affiliations:** 1 a. Guangdong Key Laboratory of Pathogen Detection for Emerging Infectious Disease Response, b. Workstation for Emerging Infectious Disease Control and Prevention, Guangdong Center for Disease Control and Prevention, Guangzhou, 511430 China; 2 School of Public Health, Southern Medical University, Guangzhou, 510515 China; 3 Sun Yat-Sen Memorial Hospital, Sun Yat-Sen University, Guangzhou, 510120 China

**Keywords:** Influenza, Seasonal characteristics, Concentration Ratio, Seasonal Index, Circular Distribution, Non-pharmaceutical Intervention (NPI)

## Abstract

**Background:**

Since the Non-pharmaceutical Intervention (NPI) by COVID-19 emerged, influenza activity has been somewhat altered.

**Objectives:**

The aim of this study was to explore changes in influenza activities in the context of COVID-19 based on the sentinel hospitals/units in Guangdong, southern China.

**Methods:**

The surveillance data in influenza-like illness (ILI) were collected from 21 cities in Guangdong between September 2017 and August 2021, while 43 hospitals/units were selected to analyze the predominant types of influenza, population characteristics, and seasonal features by three methods (the concentration ratio, the seasonal index, and the circulation distribution), based on a descriptive epidemiological approach.

**Results:**

During the four consecutive influenza seasons, a total of 157345 ILIs were tested, of which 9.05% were positive for influenza virus (n = 14238), with the highest positive rates for both IAV (13.20%) and IBV (5.41%) in the 2018–2019 season. After the emergence of COVID-19, influenza cases decreased near to zero from March 2020 till March 2021, and the dominant type of influenza virus changed from IAV to IBV. The highest positive rate of influenza existed in the age-group of 5 ~  < 15 years in each season for IAV (*P* < 0.001), which was consistent with that for IBV (*P* < 0.001). The highest annual positive rates for IBV emerged in eastern Guangdong, while the highest annual positive rates of IAV in different seasons existed in different regions. Furthermore, compared with the epidemic period (ranged from December to June) during 2017–2019, the period ended three months early (March 2020) in 2019–2020, and started by five months behind (April 2021) during 2020–2021.

**Conclusion:**

The highest positive rates in 5 ~  < 15 age-group suggested the susceptible in this age-group mostly had infected with infected B/Victoria. Influenced by the emergence of COVID-19 and NPI responses, the epidemic patterns and trends of influenza activities have changed in Guangdong, 2017–2021.

**Supplementary Information:**

The online version contains supplementary material available at 10.1007/s44197-023-00134-z.

## Introduction

Influenza (flu) is an acute respiratory infectious disease due to the influenza virus, which often causes a series of local outbreaks and seasonal epidemics, then resulting in socio-economic burden [1]. Influenza-like illness (ILI), including influenza infection, is an important public health concern, but the timing and peak intensity vary considerably from season to season and regionally [2]. Since the emergence of Coronavirus Disease 2019 (COVID-19) at the end of 2019, it has had a great impact not only on human health but also on the spread of influenza worldwide by the Non-pharmaceutical Intervention (NPI). In South Korea, the proportion of ILI in general outpatient clinics decreased to 49.8‰ in the 2019–2020 influenza season, in contrast, during 2016–2019 influenza seasons the rates of ILIs had maintained at the level of 71.9‰ to 86.2‰ [3]. Regarding China, in Hubei, during the emergency response to COVID-19 (Feb ~ Mar, 2020), the positive rates of influenza A virus (IAV) and influenza B virus (IBV) in ILIs were 4.17% and 0.29%, respectively, which were significantly lower than those in 2015–2019 (*P*_*IAV*_ < 0.001, *P*_*IBV*_ < 0.001) [4]. Additionally, in Zhenjiang of Zhejiang, the weekly average number of influenza cases between the 5th and 18th weeks in 2020 decreased by 97.5% compared with the first 4 weeks [5].

Continuous and systematic influenza surveillance could keep track of epidemic trends, predominant strains, antigenic variations and sensitivity of antiviral drugs. A previous study [6] found that the results of two methods (the concentration ratio and the circular distribution) were highly correlated (*r* = 0.905, *P* < 0.001), of which the peak of influenza virus activities appeared from October to March of following year during 2009–2018 in Qinghai. To learn the characteristics of influenza activities in Guangdong, we performed an epidemiological analysis by three methods (the concentration ratio, the seasonal index, and the circulation distribution), and explored the patterns of influenza epidemic in the context of COVID-19 based on the sentinel surveillance from 2017 till 2021.

## Materials and Methods

### Data Collection

This study is a retrospective observational study using virological surveillance of ILI. All ILI data were obtained from the National Influenza Surveillance Network (NISN, https://10.249.6.18:8881/ cdc/), including items age, collecting date, date of onset, gender, region, and virological test results of respiratory specimens. Forty-three medical sentinel hospitals/units were selected (including 30 in municipal-level regions, 13 in district-level or county-level regions), where the regions covered Chaozhou (2), Dongguan (5), Foshan (5), Guangzhou (5), Heyuan (3), Huizhou (2), Jiangmen (1), Jieyang (1), Maoming (1), Meizhou (1), Qingyuan (1), Shantou (1), Shanwei (1), Shaoguan (3), Shenzhen (2), Yangjiang (1), Yunfu (3), Zhanjiang (1), Zhaoqing (1), Zhongshan (1) and Zhuhai (2). The study period included four consecutive influenza seasons, ranging from September 2017 to August 2021.

### Epidemiological Definition

Epidemiological definitions included, (i) ILI: a case had body temperature ≥ 38 ℃, accompanied with either cough or sore throat, but a lack of molecular detection; (ii) Influenza case: an ILI tested positive for nucleic acid of influenza virus; (iii) Positive rate (PR): PR was influenza-virus-positive rate in specimen of ILIs, in both IAV and IBV.

### Region Classification

Guangdong is located in the southern China, which is classified into four regions based on geographical and cultural features. The four regions include the Rearl River Delta, the Eastern Guangdong, the Western Guangdong and the Northern Guangdong. Specifically, the pearl river delta region has nine cities, covering Guangzhou, Shenzhen, Huizhou, Foshan, Dongguan, Zhaoqing, Jiangmen, Zhongshan and Zhuhai; The Estern Guangdong region has four cities, covering Chaozhou, Jieyang, Shantou and Shanwei; The Western Guangdong region has three cities, including Maoming, Yangjiang and Zhanjiang; The North Guangdong region has five cities, covering Heyuan, Meizhou, Qingyuan, Shaoguan and Yunfu.

### Concentration Ratio

The concentration ratio (short for *CR*) [7] is a algorithm that comprehensively measures the tendency of influenza concentration by calculating the monthly distribution of influenza cases in each influenza season and labeling the seasonality. A series of computational formulas included, $r_{i} = f_{i} /N$, $C = \sqrt {R_{x}^{2} + R_{y}^{2} }$, $R_{x} = \frac{{r_{2} + r_{6} - r_{8} - r_{12} }}{2} + \frac{{\sqrt 3 \left( {r_{3} + r_{5} - r_{9} - r_{11} } \right)}}{2} + \left( {r_{4} - r_{10} } \right)$, ${R}_{y}=\frac{{r}_{3}+{r}_{11}-{r}_{5}-{r}_{9}}{2}+\frac{\sqrt{3}({r}_{2}+{r}_{12}-{r}_{6}-{r}_{8})}{2}+({r}_{1}-{r}_{7})$; where ${f}_{i}$ is the number of influenza cases in the i^th^ month, *N* is the cumulative number of influenza cases throughout the whole influenza season, and *R* describes the dispersion. The degree of concentration was divided into six stages by *CR* values, criteria were as following, 0.9 ≤ *CR* ≤ 1 for very high concentration, 0.7 ≤ *CR* < 0.9 for high concentration, 0.5 ≤ *CR* < 0.7 for moderate concentration, 0.3 ≤ *CR* < 0.5 for low concentration, 0 < *CR* < 0.3 for minimal concentration and *CR* = 0 for evenly distributed.

### Seasonal Index

The seasonal index (short for *SI*) [8] is a statistical method, in which the seasonal pattern of influenza is shown as the ratio of influenza cases per month to the monthly average number of influenza cases in each influenza season. The calculation formula is *SI* = *A*/*B* × 100%, where *A* is the number of influenza cases per month and *B* is the monthly average number of influenza cases throughout the whole influenza season. A month when its *SI* value is greater than 100% is considered an epidemic period of influenza, otherwise it is non-seasonal one.

### Circular Distribution

The Circular Distribution (short for *CD*) [6] is a method for processing periodic circular data, in which an influenza season was treated as a circle, the onset time was converted into an angle and the periodic data were transformed into linear data by trigonometric functional transformation. A set of equations included as following, $X=\frac{\sum {f}_{i}\mathit{cos}{\alpha }_{i}}{\sum {f}_{i}}$, $Y=\frac{\sum {f}_{i}\mathit{sin}{\alpha }_{i}}{\sum {f}_{i}}$, $\gamma =\sqrt{{X}^{2}+{Y}^{2}}$, $\mathit{cos}\overline{\alpha }=X/\gamma$, $\mathit{sin}\overline{\alpha }=Y/\gamma$, $s = 180^{o} /\pi \sqrt { - 2\ln \gamma } = 57.3^{o} \sqrt { - 2\ln \gamma }$. ; where ${f}_{i}$ is the number of influenza cases in the i^th^ month, *γ* describes the discrete trend, $\overline{a }$ is the mean angle and *s* is the standard deviation for $\overline{a }$.

The Rayleigh test (*Z* test) was used to test the presence of the mean angle. The formula is $Z=N{\gamma }^{2}$, where *N* is the number of influenza cases in the whole influenza season; if $Z>{Z}_{0.05}=2.996$, the mean angle has statistical significance. The period of influenza epidemic ($\overline{a }\pm s$) was predicted as *N* > 100.

### Statistical Analysis

Excel 2019 was used to deal with original data and draw figures. Data were analyzed by using SPSS v.24 (SPSS Inc., Chicago, IL). The chi-square (${\mathcal{X}}^{2}$) test was performed for categorical variables with significance level at two-tailed *P* < 0.05. The statistical analyses on data include the indicators related to above three methods (CR, SI and CD).

## Results

### General Characteristics

**Fig. 1:**
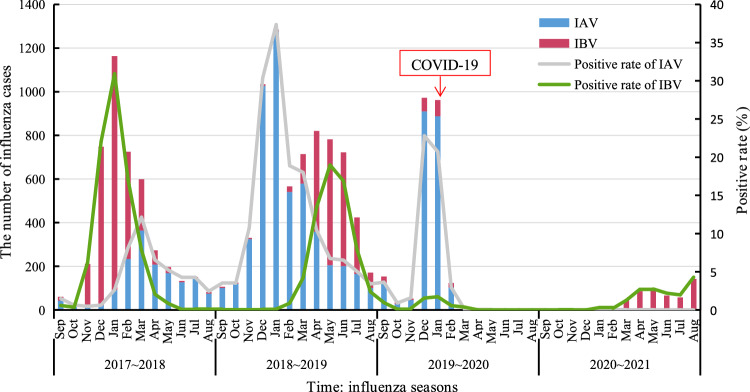
The influenza activities in Guangdong, 2017–2021

The number of ILI specimens collected for PCR testing during the four influenza seasons accounted for 23.91% (37,623), 24.15% (37,997), 26.67% (41,969) and 25.27% (39,756), respectively. The ratio of male-to-female was 1.30:1 (88 864/68 481) and the median age was 9 year-old (Interquartile range 3–34 year-old). Through PCR testing, a total of 14 238 influenza cases were sorted out, with 9.05% of positive rate. The annual positive rates showed an upward trend from 11.59% in the 2017–2018 influenza season to 18.58% in the 2018–2019 season, and then decreased to 5.51% and 1.28% in the 2019–2020 and the 2020–2021 seasons, respectively. These presented a tendency in positive rates decreasing during 2019–2021 seasons compared to the 2017–2019 seasons, even closing to zero from March 2020 to March 2021, on the back of remaining stable in the number of samples tested (Fig. 1).

A comparison of positive rates for influenza A infection over the 4 years indicated that the highest positive rate of IAV existed in the 2018–2019 season (13.16%), followed by the 2019–2020 season (5.00%) and the 2017–2018 season (4.01%), and the lowest in the 2020–2021 season (only 0.02%) (${\mathcal{X}}^{2}$=5941.86, *P* < 0.001). Unlike those, the highest positive rate of IBV emerged in the 2017–2018 season (7.57%), followed by the 2018–2019 season (5.41%) and the 2020–2021 season (1.27%), and the lowest in the 2019–2020 season (only 0.50%) (${\mathcal{X}}^{2}$=3594.92, *P* < 0.001) (Table 1).

**Table 1:** Characteristics of influenza in ILI based on the sentinel survey, 2017–2021 [n (%)]

Item	2017–2018	2018–2019	2019–2020	2020–2021	Total	Statistic	
	(N = 37,623)	(N = 37,997)	(N = 41,969)	(N = 39,756)	(N = 157,343)	${\mathcal{X}}^{2}$	*P*
Gender							
Male	2449 (11.43%)	3837 (18.04%)	1299 (5.43%)	288 (1.30%)	7873 (8.86%)	4319.10	< 0.001
Female	1908 (11.78%)	3220 (19.25%)	1011 (5.60%)	221 (1.26%)	6360 (9.29%)	3720.76	< 0.001
Age group							
< 5	1097 (6.87%)	1625 (11.32%)	410 (2.94%)	49 (0.33%)	3181 (5.39%)	1905.34	< 0.001
5 ~ < 15	1676 (23.59%)	2205 (27.68%)	943 (12.65%)	223 (3.41%)	5047 (17.36%)	1670.81	< 0.001
15 ~ < 25	448 (14.76%)	882 (24.57%)	365 (9.77%)	58 (1.53%)	1753 (12.39%)	861.87	< 0.001
25 ~ < 60	844 (11.15%)	1945 (22.90%)	427 (4.22%)	164 (1.71%)	3380 (9.45%)	2607.98	< 0.001
≥ 60	292 (7.39%)	400 (11.13%)	165 (2.46%)	15 (0.29%)	872 (4.50%)	712.70	< 0.001
Region							
PRD	2264 (11.25%)	3386 (18.00%)	1061 (4.78%)	182 (0.90%)	6893 (8.47%)	808.78	< 0.001
EG	580 (11.20%)	1027 (19.10%)	367 (6.74%)	188 (3.33%)	2162 (9.99%)	4101.88	< 0.001
WG	294 (10.80%)	749 (18.80%)	160 (4.97%)	17 (0.38%)	1220 (8.51%)	1812.82	< 0.001
NG	1219 (12.70%)	1895 (19.20%)	722 (6.51%)	122 (1.29%)	3958 (9.90%)	939.11	< 0.001
PCR test							
IAV	1510 (4.01%)	5000 (13.20%)	2100 (5.00%)	6 (0.02%)	8616 (5.48%)	5941.86	< 0.001
IBV	2847 (7.57%)	2057 (5.41%)	210 (0.50%)	503 (1.27%)	5617 (3.57%)	3594.92	< 0.001
Mix	3 (0.01%)	1 (0.003%)	1 (0.002%)	0 (0.00%)	5 (0.003%)	–	–
Sum up	4360 (11.59%)	7058 (18.58%)	2311 (5.51%)	509 (1.28%)	14238 (9.05%)	6940.69	< 0.001

*PRD* Pearl River Delta, *EG* Eastern Guangdong, *WG* Western Guangdong, *NG* Northern Guangdong, *IAV* Influenza A virus, *IBV* Influenza B virus, *Mix*  more one type/subtype, including IAV or IBV

#### Changes in the Dominant Type of Influenza Virus

Of 14 238 influenza cases including 4 360 for the 2017–2018 season, 7 058 for the 2018–2019 season, 2 311 for the 2019–2020 season and 509 for the 2020–2021 season, IAV accounted for 60.51% (n = 8616). As shown in Table 1, in the 2017–2018 season, the positive rate of IAV was significantly lower than that of IBV (${\mathcal{X}}^{2}$=387.93, *P* < 0.001), when 65.30% (n = 2847) were IBV with B/Yamagata predominating (52.87%) over B/Victoria (11.58%), besides, 34.63% (n = 1510) were IAV, of which 32.36% were ascribed to H1N1pdm, and the remaining 2.25% being H3N2. In the 2018–2019 season, the positive rate of IAV was significantly higher than that of IBV (${\mathcal{X}}^{2}$=1124.45, *P* < 0.001), when 70.84% were IAV with H1N1pdm dominating (51.88%) over H3N2 (18.94%), in addition, 29.14% were IBV, of which 28.97% were ascribed to one lineage B/Victoria, except for 0.17% being B/Yamagata. Similarly, the positive rate of IAV was significantly higher than that of IBV (${\mathcal{X}}^{2}$=1505.67, *P* < 0.001) in the 2019–2020 season, while 90.87% were IAV with H3N2 dominating (73.13%) over H1N1pdm (17.70%), besides, 9.09% were IBV, of which only B/Victoria were detected. However, in the 2020–2021 season, the detected B/Victoria (98.23%) dominated over B/Yamagata (0.59%). These represented alternately circulating among the four types/subtypes, meanwhile, after the emergence of COVID-19, the dominant type of influenza virus changed from IAV to IBV (Table 2).

**Table 2:** Positive rates of different types of influenza virus, Guangdong [n (%)]

Influenza season	IAV (n, %)		IBV (n, %)		Statistic	
	H3N2	H1N1pdm	B/Victoria	B/Yamagata	${\mathcal{X}}^{2}$	*P*
2017–2018	98 (0.26%)	1411 (3.75%)	505 (1.34%)	2305 (6.13%)	387.93	< 0.001
2018–2019	1337 (3.52%)	3662 (9.64%)	2045 (5.38%)	12 (0.03%)	1124.45	< 0.001
2019–2020	1690 (4.03%)	409 (0.97%)	210 (0.50%)	0 (0.00%)	1505.67	< 0.001
2020–2021	0 (0.00%)	0 (0.00%)	500 (1.26%)	3 (0.01%)	482.22	< 0.001

*IAV* Influenza A virus, *IBV* Influenza B virus

#### Gender Distribution

The positive rate of influenza virus in female was 9.29% (6361/68481), which was higher than that in male (8.86%, 6361/88864) (${\mathcal{X}}^{2}$=8038.89, *P* < 0.001). In addition, compared the annual positive rates of influenza viruses by gender, there were no statistically difference in both male and female in the annual positive rates for both IAV and IBV for each season. The maximum positive rates for both IAV and IBV in boy and girl existed in the 2018–2019 season and the 2017–2018 season, respectively (Table S1).

#### Age-group Distribution

School children in the 5 ~  < 15 age-group shared the highest positive rates for influenza virus (17.36%), followed by the 15 ~  < 25 age-group(12.39%) and 25 ~  < 60 age-group (9.45%). By comparing the annual positive rates by age-groups, the highest positive rate of influenza virus existed in the 5 ~  < 15 age-group, ranging from 3.41% to 27.68%; in contrast, the lowest positive rates emerged in both 0 ~  < 5 age-group and ≥ 60 age-group, covering 0.33%–11.32% and varying 0.29%–11.13%, respectively. In addition, the annual positive rates of both IAV and IBV existed in different age-groups, the highest positive rate was in the age-group of 5 ~  < 15 age-group in each influenza season for IAV (*P* < 0.001), which was consistent with that for IBV (*P* < 0.001). The maximum annual positive rates for both IAV and IBV appeared in 5 ~  < 15 age-group existed in the 2018–2019 season (18.45%) and the 2017–2018 season (18.17%), respectively (Table S2).

#### Region Distribution

Comparing the positive rates of different four regions, the lowest positive rate emerged in Western Guangdong for both IAV and IBV in each influenza season (*P* < 0.001), the annual highest positive rate of IAV emerged in the Pearl River Delta region (4.05%), Eastern Guangdong (13.7%) and Northern Guangdong (5.82%) in the 2017–2018, the 2018–2019 and the 2019–2020 seasons, respectively. For IBV, the annual highest positive rate existed in Eastern Guangdong from 2017 to 2021, except for the 2018–2019 season (Table S3).

### Seasonal Characteristics of Influenza

#### Concentration Ratio

Based on the monthly number of influenza cases from 2017 to 2021 in Guangdong, the concentration of influenza activities in each influenza season was analyzed, shown in Table 3. Four influenza seasons represented concentrating, as the values of concentration ratio in the 2019–2021 influenza season (*CR*_2019-2020_ = 0.85) were higher than that in the 2017–2019 seasons, whereas there were moderate concentrations in both the 2017–2018 season and the 2020–2021 seasons (*CR*_2017-2018_ = 0.57, *CR*_2020-2021_ = 0.58), of which the high concentration was in the 2019–2020 season.

**Table 3:** Concentration ratio (*CR*) of influenza activity, Guangdong

Item	2017–2018	2018–2019	2019–2020	2020–2021
${\mathrm{R}}_{\mathrm{x}}$	0.39	0.07	0.83	− 0.58
${\mathrm{R}}_{\mathrm{y}}$	− 0.41	− 0.31	− 0.17	− 0.05
$CR$	0.57^a^	0.32 ^b^	0.85^c^	0.58^a^

a, b and c denote moderate, low and high concentration, respectively

#### Seasonal Index

The seasonal index (*SI*) was obtained by calculating the ratio of influenza cases per month to the average number of influenza cases in each influenza season. The *SI* values (%) covered from 0 to 320.09, then based on *SI* > 100%, the influenza epidemic periods were sorted out, including December 2017 to March 2018, December 2018 to January 2019, March 2019 to June 2019, December 2019 to January 2020 and March 2021 to June 2021, respectively, shown in Table 4. Compared with the 2017–2020 season, the influenza epidemic period was delayed in 2020–2021 (December *vs*. March 2021).

**Table 4:** Seasonal index (*SI*) of influenza activity, Guangdong

Influenza season	*SI* (%)											
	Sep	Oct	Nov	Dec	Jan	Feb	Mar	Apr	May	Jun	Jul	Aug
2017–2018	16.51	8.53	58.07	205.87*	320.09*	199.54*	164.86*	75.14	53.94	35.50	40.46	21.47
2018–2019	17.34	20.57	55.43	175.12*	217.63*	96.23	121.39*	139.42*	132.96*	122.75*	72.09	29.07
2019–2020	79.45	15.58	27.00	504.72*	499.52*	63.87	8.31	1.04	0.00	0.00	0.00	0.52
2020–2021	0.00	2.36	2.36	2.36	25.93	16.50	106.09*	202.75*	216.90*	155.60*	134.38*	334.77*

*Denotes *SI* > 100%

#### Circular Distribution

According to the number of influenza cases, the epidemic period for each influenza season was calculated separately by using the circular distribution method, as the epidemic periods shown in Table 5. These results presented that the epidemic period in the 2017–2019 influenza season was from November to June of the following year (*Z*_2017-2018_ = 399.29, *Z*_2018-2019_ = 391.28, $P$<0.05), the period in the 2019–2020 season covered from December to March of the following year (*Z*_2019-2020_ = 1084.36, $P$<0.05), and the period lasted from April to August in the 2020–2021 season (*Z*_2020-2021_ = 21.86, $P$<0.05).

**Table 5:** The mean angles ($\overline{a }$) and epidemic periods of influenza, Guangdong

Influenza season	$\overline{a }$	$s$	$\gamma$	$Z$	$P$	Epidemic period
2017–2018	182.56	88.59	0.30	399.29	< 0.05	November 16th–June 2nd
2018–2019	173.31	97.44	0.24	391.28	< 0.05	December 4th–June 2nd
2019–2020	151.00	49.84	0.68	1084.36	< 0.05	December 12th–March 23rd
2020–2021	323.66	101.66	0.21	21.86	< 0.05	April 14th–August 31st

$\overline{a }$*,* $s$ and $\gamma$ denote the mean angle, the standard deviation of mean angle and the discrete trend, respectively

## Discussion

The overall positive rate of influenza in ILI was 9.05% (14 238/157 343) from 2017 till 2021 in this study, and the annual positive rates of influenza virus in ILIs showed a decreasing tendency during 2018–2021 (15.58%/ 5.51%/ 1.28%, annually respectively), which indicated that influenza virus activity during 2019–2021 was significantly lower than that in the previous two influenza seasons. Similarly, the decreases of PRs also occurred in Canada during 2020–2021, with IAV and IBV being only 1.5‰ and 2.8‰ times of 2014–2020, respectively [9]. During the COVD-19 pandemic, to inhibit the spread of SARS-CoV-2 by NPI, the Guangdong health administration implemented three levels of public health emergency responses, in which the first phase started on 23 January 2020 and lasted until 23 February 2020, the second phase varying from 24 February to 8 May 2020, and the third phase covered from 9 May 2020 to 31 August 2021. Based on the substantial decline in influenza virus activity during 2019–2021, the following reasons have been suggested. Firstly, both influenza virus and SARS-CoV-2 belong to respiratory infectious disease pathogens and have the same mode of transmission, therefore the general precautions (including the wearing of masks and hand washing, etc.) for COVID-19 prevention could reduce the frequency of influenza virus infections [10]. Secondly, fewer reports of influenza cases during the COVID-19 epidemic were due to both that people altered their health-seeking behavior and influenza is a self-limiting illness. Thirdly, online teaching and other methods reduced mass gatherings, which could hold down the influenza outbreaks in school. Finally, the viral interference between SARS- CoV-2 and influenza virus might be one of reasons for low influenza virus circulation [11, 12].

Previous studies in southern China showed that seasonal influenza epidemics mainly were attributed to IAV subtypes of A/H1N1pdm and A/H3N2 in addition to IBV (B/Victoria and B/Yamagata) [13, 14]. Four types/subtypes alternately circulated during the four consecutive seasons in this study, similarly report occurred in Western Saudi Arabia [15]. Moreover, IAV dominating in Guangdong was line with a previous report in Iran [16]. However, after the emergence of COVID-19, the dominant type of influenza virus was changed from IAV into IBV, which is consistent with that of the overall situation in China [17]. Since then, a novel evolutionary branch (V1A.3a.2) evolved from B/Victoria gene dominated in southern China [18] of which the travel restrictions in NPI during the COVID-19 pandemic had affected the spread of other types of influenza strains in different regions [19].

The female than male in this study accounted for a higher positive rate (9.29%/ 8.86%), which were different from those in a previous study (12.4% *vs* 23.0%) [20]. School children in the 5 ~  < 15 age-group had the highest positive rates for influenza virus (17.36%), followed by the 15 ~  < 25 (12.39%), which was lower than the positive rate in Georgia in this age-group (48.2%) [21]. Similar to the results in this study, the patients infected with B/Victoria in Italy were mainly aged 5 ~  < 15 age-group (51.7%) [22]. The highest positive rates in 5 ~  < 15 age-group indicated the susceptible mostly had infected with infected B/Victoria, which is the dominant lineage and takes an absolute epidemic advantage in the 2020–2021 season, regardless of the NPI reducing the risk of SARS-COV-2 transmission during the pandemic. In addition, younger than 5 years old group had the largest proportion in ILI specimens (37.49%), while the positive rate in this group was only 5.39%, which may be influenced by other non-influenza viruses. Adam K et al. reported that in the < 5 years old, the positive rates of adenovirus, rhinovirus and respiratory syncytial virus were higher than that for influenza virus [23], which suggested that this population was susceptible to multiple respiratory pathogens. Meanwhile, personnel coordination and the quality of samples may also be one of the reasons for the low positive rate in this age-group of < 5 years old.

Analysis on epidemic pattern of infectious diseases usually was adopted with different epidemiological methods, of which three methods including CR, SI and CD in this study [6–8]. The method CR represents a concentration trend based on algebraic cumulative calculation throughout whole season, while the method SI is a ratio based on the specific measured value to monthly average value, revealing the seasonal pattern, and the method CD uses circular periodic in trigonometric function to assess statistical distribution, projecting the epidemic period.

In southern China (including Guangdong), the epidemic periods of influenza in this study began in November across six months in the 2017–2019 influenza seasons, but the period in 2019–2020 ended three months earlier than that in 2018–2019, while the period in 2020–2021 was from April till August. The epidemic characteristic changes might relate with the prevention and control measures to COVID-19 (such as human mobility, social distance and personal hygiene), but it is still worth exploring the main causes for the changes of influenza epidemic in the context of the COVID-19 pandemic. In conclusion, the results in this study presented the changes in the epidemic patterns and trends of influenza activities impacted by NPI based on the sentinel surveillance.

## Supplementary Information

Below is the link to the electronic supplementary material.

Supplementary file1 (DOCX 23 KB)

## Data Availability

All data and materials are available upon the contact with the authors.

## Abbreviations

COVID-19: Coronavirus Disease 2019
Flu: Influenza
NPI: Non-pharmaceutical Intervention
IAV: Influenza A Virus
IBV: Influenza B Virus
CR: Concentration Ratio
SI: Seasonal Index
CD: Circular Distribution
