# Supplementary material for: Changes in Influenza Activities Impacted by NPI Based on 4-Year Surveillance in China: Epidemic Patterns and Trends
Source: J Epidemiol Glob Health. 2023 Aug 3;13(3):539–46. doi: 10.1007/s44197-023-00134-z (PMC10468473; doi:10.1007/s44197-023-00134-z)
Supplement: Supplementary file 1 — Supplementary file1 (DOCX 23 KB) [file 44197_2023_134_MOESM1_ESM.docx]

**Table S1.** Positive rates of influenza virus by PCR in male and female, Guangdong [n (%)]

| Type | 2017-2018 | |  | 2018-2019 | |  | 2019-2020 | |  | 2020-2021 | |
| --- | --- | --- | --- | --- | --- | --- | --- | --- | --- | --- | --- |
|  | male (N=21426) | female (N=16197) |  | male (N=21272) | female (N=16725) |  | male (N=23928) | female (N=18039) |  | male (N=22237) | female (N=17519) |
| IAV | 825  (3.85%) | 685  (4.23%) |  | 2692 (12.66%) | 2308 (13.80%) |  | 1169 (4.89%) | 130  (5.16%) |  | 4  (0.02%) | 2  (0.01%) |
| IBV | 1624 (7.58%) | 1223  (7.55%) |  | 1145 (5.38%) | 912  (5.45%) |  | 931  (0.54%) | 80  (0.44%) |  | 284  (1.28%) | 219  (1.25%) |
| Total | 2449 (11.43%) | 1908 (11.78%) |  | 3837 (18.04%) | 3220 (19.25%) |  | 1299 (5.43%) | 1011  (5.60%) |  | 288  (1.30%) | 221  (1.26%) |

Note: IAV=Influenza A virus, IBV=Influenza B virus.

**Table S2.** Positive rates of influenza virus by PCR in different age-groups, Guangdong [n (%)]

| Type | 2017-2018 | | | | |  | 2018-2019 | | | | |  | 2019-2020 | | | | |  | 2020-2021 | | | | |
| --- | --- | --- | --- | --- | --- | --- | --- | --- | --- | --- | --- | --- | --- | --- | --- | --- | --- | --- | --- | --- | --- | --- | --- |
|  | ＜5  (N=15960) | 5~＜15  (N=7106) | 15~＜25  (N=3035) | 25~＜60  (N=7569) | ≥60  (N=3953) |  | ＜5  (N=14353) | 5~＜15  (N=7967) | 15~＜25  (N=3590) | 25~＜60  (N=8494) | ≥60  (N=3593) |  | ＜5  (N=13964) | 5~＜15  (N=7456) | 15~＜25  (N=3735) | 25~＜60  (N=10115) | ≥60  (N=6697) |  | ＜5  (N=14706) | 5~＜15  (N=6536) | 15~＜25  (N=3789) | 25~＜60  (N=9603) | ≥60  (N=5122) |
| IAV | 505  (3.16%) | 385  (5.42%) | 123  (4.05%) | 395  (5.22%) | 102  (2.58%) |  | 1230  (8.57%) | 1470  (18.45%) | 597  (16.63%) | 1345  (15.83%) | 358  (9.96%) |  | 364  (2.61%) | 864  (11.59%) | 340  (9.10%) | 373  (3.69%) | 159  (2.37%) |  | 3  (0.02%) | 1  (0.02%) | 0  (0.00%) | 2  (0.02%) | 0  (0.00%) |
| IBV | 592  (3.71%) | 1291  (18.17%) | 325  (10.71%) | 449  (5.93%) | 190  (4.81%) |  | 395  (2.75%) | 735  (9.23%) | 285  (7.94%) | 600  (7.06%) | 42  (1.17%) |  | 46  (0.33%) | 79  (1.06%) | 25  (0.67%) | 54  (0.53%) | 6  (0.09%) |  | 46  (0.31%) | 222  (3.40%) | 58  (1.53%) | 162  (1.69%) | 15  (0.29%) |
| Total | 1097  (6.87%) | 1676  (23.59%） | 448  (14.76%) | 844  (11.15%) | 292  (7.39%) |  | 1625  (11.32%) | 2205  (27.68%) | 882  (24.57%) | 1945  (22.90%) | 400  (11.13%) |  | 410  (2.94%) | 943  (12.65%) | 365  (9.77%) | 427  (4.22%) | 165  (2.46%) |  | 49  (0.33%) | 223  (3.41%) | 58  (1.53%) | 164  (1.71%) | 15  (0.29%) |

Note: IAV=Influenza A virus, IBV=Influenza B virus.

**Table S3.** Positive rates of influenza virus by PCR in different regions, Guangdong [n (%)]

| Type | 2017-2018 | | | |  | 2018-2019 | | | |  | 2019-2020 | | | |  | 2020-2021 | | | |
| --- | --- | --- | --- | --- | --- | --- | --- | --- | --- | --- | --- | --- | --- | --- | --- | --- | --- | --- | --- |
|  | PRD  (N=20129) | EG  (N=5177) | WG  (N=2722) | NG  (N=9595) |  | PRD  (N=18767) | EG  (N=5386) | WG  (N=3978) | NG  (N=9866) |  | PRD  (N=22214) | EG  (N=5448) | WG  (N=3219) | NG  (N=11086) |  | PRD  (N=20263) | EG  (N=5640) | WG  (N=4422) | NG  (N=9431) |
| IAV | 815  (4.05%) | 197  (3.81%) | 141  (5.18%) | 357  (3.72%) |  | 2362  (12.60%) | 736  (13.70%) | 540  (13.60%) | 1362  (13.80%) |  | 1010  (4.55%) | 300  (5.51%) | 145  (4.50%) | 645  (5.82%) |  | 2  (0.01%) | 0  (0.00%) | 0  (0.00%) | 4  (0.04%) |
| IBV | 1449  (7.20%) | 383  (7.40%) | 153  (5.62%) | 862  (8.98%) |  | 1024  (5.46%) | 291  (5.40%) | 209  (5.25%) | 533  (5.40%) |  | 51  (0.23%) | 67  (1.23%) | 15  (0.47%) | 77  (0.69%) |  | 180  (0.89%) | 188  (3.33%) | 17  (0.38%) | 118  (1.25%) |
| Total | 2264  (11.25%) | 580  (11.20%) | 294  (10.80%) | 1219  (12.70%) |  | 3386  (18.00%) | 1027  (19.10%) | 749  (18.80%) | 1895  (19.20%) |  | 1061  (4.78%) | 367  (6.74%) | 160  (4.97%) | 722  (6.51%) |  | 182  (0.90%) | 188  (3.33%) | 17  (0.38%) | 122  (1.29%) |

Note: PRD=Pearl River Delta, EG=Eastern Guangdong, WG=Western Guangdong, NG=Northern Guangdong.
